# Refined Lactulose Hydrogen Breath Test for Small Intestinal Bacterial Overgrowth Subgrouping Irritable Bowel Syndrome With Low and High Breath Hydrogen

**DOI:** 10.1155/grp/5597071

**Published:** 2025-09-15

**Authors:** A. Dahlgren, P. Grybäck, H. Jacobsson, P. M. Hellström

**Affiliations:** ^1^Department of Medical Sciences, Uppsala University, Uppsala, Sweden; ^2^Department of Hospital Physics and Nuclear Medicine, Karolinska University Hospital, Solna, Sweden

**Keywords:** IBS, lactulose, microbiota, small intestinal bacterial overgrowth

## Abstract

**Background:** Small intestinal bacterial overgrowth (SIBO) is suggested in irritable bowel syndrome (IBS). Our primary aim was to define a discriminating threshold for a positive lactulose hydrogen breath test (LHBT) in SIBO. As a secondary aim, IBS was subdivided into SIBO and non-SIBO groups.

**Methods:** LHBT performed in 206 subjects, 74 healthy subjects, 39 SIBO patients with intestinal lesions, 77 IBS patients, and 16 nonhydrogen producers. Using scintigraphy and LHBT, orocecal transit time was set to 80 min. Peak hydrogen levels were compared between the groups. Values are mean and 95% confidence interval.

**Results:** Using an 80-min orocecal cutoff time, LHBT in healthy subjects had peak values of 8 (6–9) ppm and SIBO 38 (31–45) ppm (*p* < 0.0001). The diagnostic cutoff 20 ppm verified a sensitivity of 77% and specificity of 88% and positive and negative predictions of 77% and 88%. With the same cutoff for IBS, the mean peak value was 21 (16–26) ppm (*p* < 0.0001 vs. healthy) with a sensitivity of 39% and a specificity of 78% and positive and negative predictions of 77% and 84%. Separating IBS at 20 ppm, the low-hydrogen group had a peak value of 6 (5–7) ppm (ns vs. healthy), and the high-hydrogen group had a peak of 44 (38–49) ppm (*p* < 0.0001 vs. healthy). After antibiotics, IBS with low hydrogen remained unchanged, whereas those with high hydrogen were reduced to control (*p* < 0.01).

**Conclusion:** With cutoff at 20 ppm, LHBT differentiates SIBO in patients with early high breath hydrogen peaks, subdividing IBS into non-SIBO and SIBO groups; the latter may benefit from antibiotic treatment.

**Summary**

• Lactulose hydrogen breath test is used to diagnose small intestinal bacterial overgrowth.
• An appropriate diagnostic hydrogen cutoff level of 20 ppm indicates that a substantial proportion of patients with irritable bowel syndrome have small bacterial overgrowth.
• Normalization of breath hydrogen after antibiotic treatment, along with symptom relief, suggests the bacterial overgrowth in selected cases of irritable bowel syndrome.

## 1. Introduction

Accumulating evidence supports the role of dysbiosis in irritable bowel syndrome (IBS) [1–3]. Small intestinal bacterial overgrowth (SIBO) is detected in 4%–78% of IBS cases [4]. This huge variability in prevalence may be due to ethnicity, microbiological milieu, and the employed diagnostic criteria. Noninvasive breath tests have gained popularity owing to their feasibility and speed at which a diagnosis can be made. However, bacterial cultures of aspirate from the proximal jejunum are still considered the gold standard for diagnosing SIBO [5]. However, this has been challenged because it carries a risk of sampling error if not all regions of the small bowel are sampled. In addition, there is a contamination risk of nearly 20%, and approximately 80% of the sampled bacteria are not culturable, including most hydrogen-producing species, making this diagnostic method suboptimal [5].

Different types of breath tests have been used but are hampered by the lack of a true standard for the performance and interpretation of data. Depending on the substrate used as well as the timing of readouts and diagnostic hydrogen cutoff levels, various studies have arrived at different recommendations for the optimization of the breath test for diagnosing SIBO. This has led to interactive working groups advocating essential rules for employing hydrogen breath tests for diagnostic purposes [6–8]. One of the main problems is the difficulty to set a valid cutoff for small bowel hydrogen at the same time avoiding lactulose fermentation by the cecal microflora.

The lactulose hydrogen breath test (LHBT) is clinically used as a noninvasive proxy for diagnosing SIBO. The main limiting factor is the time frame for the readout of hydrogen in the breath. Lactulose is a nondigestible, nonabsorbable disaccharide substrate for bacterial hydrogen production, which was sampled throughout the entire small intestine to identify SIBO. Under normal conditions, no significant amount of hydrogen is present in the breath until fermentation occurs in the cecum, depending on the colonization of bacteria in this region. Initially, Bond et al. [9] showed that healthy subjects had an orocecal transit time (OCTT) of 72 min, while Mathur et al. [10, 11] used a transit time of 90 min. The North American guidelines have later built a consensus for a cutoff time point of 90 min during LHBT for diagnosing SIBO [6], although European guidelines suggest that a lactulose meal can reach the colon in considerably less time in patients with a rapid OCTT [7].

Regarding diagnostic hydrogen cutoff levels, several investigators have reported different cutoff levels for SIBO, such as 5, 10, and 12 ppm [7] as well as 20 ppm [6]. Using the 20 ppm cutoff, a diagnostic agreement of 53% has been reported for small bowel cultures of ≥ 10^3^ CFU/mL [12].

This study was conducted to refine the optimal diagnostic cutoff values for LHBT. After the optimal transit time was verified by small bowel scintigraphy, diagnostic hydrogen levels were compared between healthy controls, patients with anatomical lesions or dysmotility of the small intestine predisposing to SIBO, and patients with IBS. We used these diagnostic levels for SIBO to subdivide the IBS patient group into those with and without SIBO.

## 2. Materials and Methods

### 2.1. Ethical Considerations

The need for consent to participate was deemed unnecessary by the Institutional Review Board (IRB) according to national regulations for anonymized data. The study was conducted in accordance with the principles of the Declaration of Helsinki. The study was approved by the Uppsala IRB (2022-04646-01); all patients' identities were anonymized.

### 2.2. Preparatory Scintigraphy Combined With LHBT

Thirty-one healthy subjects (14 men, 17 women, 19–77 years of age) were recruited for small bowel scintigraphy with lactulose as a second marker for transit time using the LHBT.

The studies were performed in the supine position at 8:00 AM after an overnight fast. After intravenous administration, the radioactive marker, 120 MBq ^99m^Tc-HIDA (Amersham Sorin, Saluggia, Italy), was rapidly excreted into the duodenum by the bile. Images were recorded with anterior 1-min acquisitions obtained in dynamic mode (for details, see [13]).

In the scintigraphic examinations, the regions of interest (ROIs) were outlined over the proximal duodenum and cecum, and the radioactive count rate was evaluated against background radioactivity. The time of appearance of the radioactive marker in each ROI was defined as a more than twofold increase compared to the background radioactivity within that ROI. The time points for the appearance of radioactivity in each ROI were considered the onset and endpoint of transit.

Imaging was started immediately after the administration of ^99m^Tc-HIDA. When the radionuclide accumulated in the gallbladder and central bile ducts, 10 g lactulose solution (670 mg/mL; Laktulos Meda, Meda AB, Solna, Sweden) was administered along with breath sampling and continued until the radionuclide was detected in the cecum, and transit time was measured by a more than twofold increase in hydrogen compared to prior readout (for details, see [13]).

### 2.3. LHBT

The LHBT was performed on 206 subjects, 16 of which were hydrogen nonproducers and excluded from further investigations. Hence, 190 subjects were divided into three groups according to the Rome III criteria: 74 healthy controls (36 men, 38 women, 22–79 years of age), 39 individuals considered predisposed to SIBO due to anatomical lesions such as stenosis or diverticula or other changes that promote stasis of small bowel contents (15 men, 24 women, 18–74 years of age), and 77 patients diagnosed with IBS (33 men, 44 women, 22–75 years of age; 11 constipation-dominant [IBS-C], 29 diarrhea-dominant [IBS-D], and 37 mixed type [IBS-M]) [14].

The LHBT was carried out using breath sampling equipment with an electrochemical hydrogen sensitive cell (GMI Medical Ltd, Renfrew, United Kingdom) with a resolution of 1 ppm, an accuracy of ± 2 ppm or 5% of full scale, and a linear range of 2–150 ppm.

After an overnight fast from 8:00 PM, breath sampling was performed the following morning at 8:00–10:00 AM. Two baseline end-expiratory breath samples were collected at −10 and 0 min. Thereafter, 10 g of lactulose solution (670 mg/mL; Laktulos Meda) was ingested, and breath hydrogen samples were collected every 10 min over the following 180 min. During the breath sampling period, the participants were not allowed to exercise, drink, eat, or smoke, as interference may cause falsely high hydrogen values. Subjects with lactase or sucrase deficiency, celiac disease, bile acid malabsorption, ulcerative colitis, Crohn's disease, collagenous or lymphocytic colitis, gastrointestinal cancer, or signs of maldigestion or general malabsorption were excluded from the study. Drug treatment with antibiotics, dopamine or opioid receptor agonists, proton pump inhibitors, and laxatives was discontinued at least 28 days before the LHBT was performed.

The breath hydrogen concentration of each subject was plotted as the hydrogen concentration (parts per million) against time (minutes) over the assessment period. Peak hydrogen values and the area under the curve from 0 to 80 min (AUC_0–80_) were extracted to calculate diagnostic accuracy. Additionally, to determine cutoff hydrogen values, a third-degree polynomial was applied to the breath hydrogen curves, and the cutoff was set at a three times increase of the curve rise.

### 2.4. Statistics

Statistical differences between scintigraphy and lactulose transit times were calculated using paired Student's *t*-test with the double background as the cutoff for the arrival of the marker in the cecum. A comparison between the two methods was performed using the Bland–Altman plot. Thereafter, differences between the SIBO study groups were calculated using ANOVA, with healthy controls as comparators.

To optimize the evaluation of the LHBT data, both the peak values at different time points and the total integrated breath hydrogen were determined by calculating the AUC over 0–80 min.

Validation of the peak hydrogen level as the diagnostic cutoff for SIBO was carried out as a sequential lineup of the breath hydrogen results for all study groups. To determine cutoff hydrogen values, a third-degree polynomial was applied to the breath hydrogen curves. Notches in the hydrogen curve at 15 and 20 ppm were interpreted to represent a three- and fourfold rise of the curve compared to the baseline. To validate the diagnostic performance of the LHBT, the 12, 15, and 20 ppm cutoffs were evaluated.

Receiver operating characteristics (ROCs) were employed to set cutoff points and calculate the accuracy of the LHBT. Values are presented as mean ± 95%confidence interval within parentheses or mean ± SE. Statistical significance was set at *p* < 0.05.

## 3. Results

**Figure 1:**
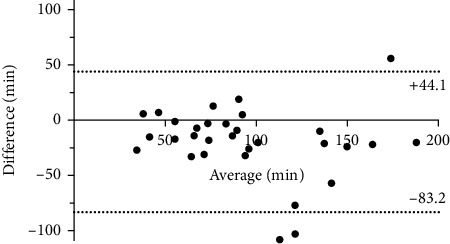
Bland–Altman plot of transit time by scintigraphy and lactulose showing the difference and 95% limits of agreement by the dashed lines. Calculated difference was outside the 95% confidence interval in three subjects.

Using scintigraphy combined with LHBT in healthy subjects, the scintigraphic transit time was 85 (70–101) min, whereas the LHBT transit time was 104 (87–121) min with no difference between the sexes. An acceptable agreement for scintigraphic and lactulose transit times was verified by the Bland–Altman plot with a bias of −19 min, showing a few assessment points outside the 95% confidence interval (Figure 1).

In the following LHBT study, the healthy control group had an OCTT of 91 (84–99) min. The OCTT of the total IBS group was 104 (97–111) min, which included the high hydrogen-producing group with possible SIBO.

After subtracting the transit time 112 (103–120) min of low hydrogen-producing IBS, data showed a mean OCTT of 91 (80–102) min for the high-hydrogen IBS group.

The lower limit of the 95% confidence interval of the LHBT transit time in healthy controls was used to set the breath time to 0–80 min as the boundary of interest for the assessment of hydrogen production in the small intestine.

**Figure 2:**
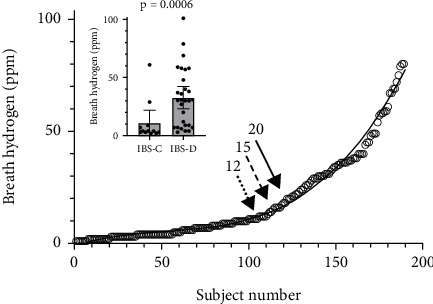
Breath hydrogen lineup of all subjects included in the study (*n* = 190). Arrows indicate the 12, 15, and 20 ppm breath hydrogen points for calculation of diagnostic accuracy. The solid line shows the third-degree polynomial of the curve. Insert shows breath hydrogen production (mean and 95% confidence interval) of subjects diagnosed with IBS-C versus IBS-D. Data indicate a curve rise by three- and fourfold at 15 and 20 ppm, respectively. Insert shows the breath hydrogen levels of patients with IBS-C as compared to IBS-D.

The LHBT data showed expected hydrogen peak values from 1 to 104 ppm in all study groups, with lactulose passing through the stomach to the cecum within the 180-min assessment time frame. There was a markedly higher hydrogen production in 20 out of 29 patients (69%) with symptoms of IBS-D as compared with IBS-C, where only 2 out of 11 (18%) displayed high hydrogen (*p* = 0.0006) (Figure 2, insert). Breath hydrogen values of all patients were adapted to the polynomial, *f*(*x*) = 0.000025*x*^3^ − 0.004*x*^2^ + 0.2641*x* − 1.207; *r*^2^ = 0.9804 showing a smooth increasing curve (Figure 2).

**Figure 3:**
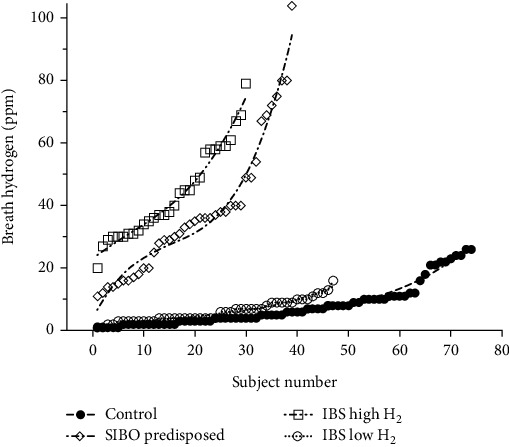
Differences in breath hydrogen between healthy controls (*n* = 74), IBS low hydrogen (*n* = 47), IBS high hydrogen (*n* = 30), and patients predisposed to SIBO (*n* = 39). The broken lines show the third-degree polynomial of each curve.

Using the 20-ppm hydrogen level as a cutoff value for diagnostic purposes, clear differences between the study groups were found. IBS patients with high hydrogen levels and those predisposed to SIBO were significantly different from IBS patients with low hydrogen levels and healthy subjects (both *p* < 0.0001). There was no statistical difference between the groups predisposed to SIBO and IBS patients with high breath hydrogen, as well as between IBS patients with low breath hydrogen and healthy controls (Figure 3).

Employing a curve-fitting approach showed differences in breath hydrogen graphs between healthy controls with the polynomial (*f*[*x*] = 0.000163*x*^3^ − 0.01202*x*^2^ + 0.3629 − 0.3487; *r*^2^ = 0.9764), IBS with low breath hydrogen (*f*[*x*] = 0.0001749*x*^3^ − 0.007753*x*^2^ + 0.252*x* + 1.172; *r*^2^ = 0.9689), IBS with high hydrogen (*f*[*x*] = 0.001683*x*^3^ − 0.03529*x*^2^ + 1.27 + 23.13; *r*^2^ = 0.9784), and those predisposed to SIBO (*f*[*x*] = 0.003749*x*^3^ − 0.1711*x*^2^ + 3.306*x* + 3.652; *r*^2^ = 0.9736).

For comparison, the integrated breath hydrogen AUC_0–80_ values showed similar outcomes as the peak values. In the healthy, AUC_0–80_ values were 206 (164–248) ppm∗80 min, while those predisposed to SIBO were 1502 (1315–1689) ppm∗80 min (*p* < 0.0001), compared to IBS with low hydrogen at 244 (199–288) ppm∗80 min (ns vs. healthy) or IBS with high hydrogen 1530 (1088–1973) ppm∗80 min (*p* < 0.0001 vs. healthy). Since the integrated hydrogen AUC did not differ from the peak levels, further validation of the cutoff levels was carried out using peak hydrogen.

**Figure 4:**
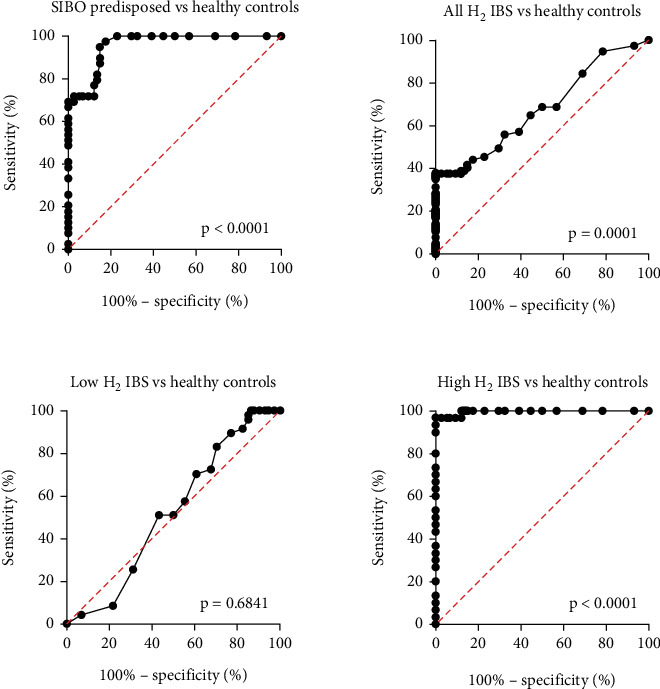
Receiver operating characteristics of the lactulose hydrogen breath test using cutoff 20 ppm, in subjects predisposed to small intestinal bacterial overgrowth (SIBO) as well as IBS, separated into low- and high-hydrogen subgroups.

ROC analysis of the LHBT was performed at different cutoff levels. First, SIBO-predisposed patients were compared to healthy controls at a 20 ppm cutoff level. This was matched to cutoff levels of 12 and 15 ppm, which showed higher sensitivity but less specificity. Next, the entire IBS group was compared to healthy controls, which showed lower sensitivity but comparable specificity, albeit with a lower likelihood ratio. By separating the IBS group at 20 ppm into high- and low-hydrogen groups, the high-hydrogen group had similar sensitivity and specificity as the SIBO group, but with a better likelihood ratio. The low-hydrogen IBS group displayed similar sensitivity, but worse specificity, compared to healthy subjects (Table 1, Figure 4).

### 3.1. SIBO in Different Patient Groups

**Figure 5:**
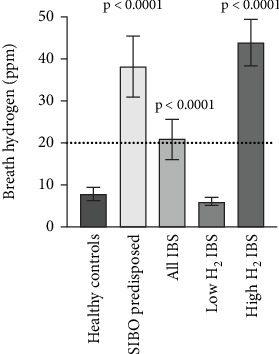
Mean breath hydrogen with 95% confidence interval of the lactulose hydrogen breath test in healthy controls and patients predisposed to SIBO, as well as irritable bowel syndrome, divided into low and high breath hydrogen. Dashed line indicates a cutoff level at 20 ppm.

The mean breath hydrogen level was 8 (6–9) ppm in healthy controls, with the highest peak at 26 ppm. In the SIBO-predisposed group, the mean breath hydrogen was 38 (31–45) ppm (*p* < 0.0001 vs. healthy), 9 of which showed breath hydrogen below the cutoff of 20 ppm and peaking at 104 ppm. In the total IBS group, the mean breath hydrogen level was 21 (16–26) ppm (*p* < 0.0001 vs. healthy). The IBS group with low hydrogen had a mean of 6 (5–7) ppm (ns vs. healthy) and the IBS group with high hydrogen 44 (38–49) (*p* < 0.0001 vs. healthy), showing peak values at 16 and 79 ppm, respectively (Figure 5).

### 3.2. Response of IBS to Antibiotics

**Figure 6:**
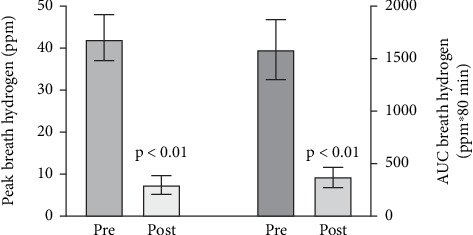
Result of antibiotic treatment on breath hydrogen in irritable bowel syndrome, monitored as peak levels (left) or AUC_0–80_ min (right); *n* = 77. Pre, before antibiotic treatment; post, after antibiotic treatment. Values are mean and 95% confidence values.

All IBS patients were given antibiotics and retested after 14 days of treatment. A majority (*n* = 28) of the patients with high breath hydrogen (*n* = 30) showed a clear reduction of breath hydrogen (Figure 6), along with improvement of abdominal distention (*n* = 28) and relief of diarrhea (*n* = 26). The IBS patients with low hydrogen showed no reduction in breath hydrogen.

## 4. Discussion

The hydrogen breath test is a widely utilized and accepted noninvasive method to diagnose SIBO because only the microbiota can produce hydrogen. The North American consensus for breath testing recommends an increase in hydrogen concentration of 20 ppm from baseline within 90 min [6]. European guidelines do not explicitly specify the diagnostic readout time points and cutoff hydrogen levels [7]. Our scintigraphy data show that lactulose is rapidly propelled to the cecum and detected by a hydrogen increase at 80 min; therefore, we chose a time frame of 0–80 min for our readings. In this way, we decreased the risk of overdiagnosing SIBO merely because of rapid transit. This resulted in a sensitivity of 39% and a specificity of 87% when diagnosing high hydrogen in IBS versus healthy controls. For comparison, employing a readout time of 60 min with a 20 ppm cutoff decreased the sensitivity to 21%, keeping the specificity at 86%. This indicates a reduced accuracy by using a shorter, for example, 60 min, diagnostic breath sampling period. Theoretically, the head of the lactulose meal may rapidly travel through the gut and reach the cecum, whereas the bulk of lactulose remains in the small intestine during fermentation in cases of SIBO. Hence, the two sources of fermentation at different sites in the gut could produce an increase in breath hydrogen, leaving the investigator with nonspecific results. However, Read and colleagues noted that when lactulose was directly administered into the cecum, it took approximately 40 min for breath hydrogen to reach 20 ppm. It is therefore possible that even if a small amount of lactulose rapidly travels to the cecum, the bulk of lactulose remaining in the small intestine is likely the main source of the early rise of hydrogen in breath [15, 16].

Our analysis, as determined from the alignment of a polynomial curve, arrived at two different cutoff levels, 15 and 20 ppm, at which point small indents in the curve markedly increased the derivative of the polynomial, suggesting increased hydrogen production. The 12-ppm readout time point was included for comparative reasons. Calculations of the diagnostic accuracy with these cutoff points showed that the sensitivity of the breath test was improved by using the 20-ppm cutoff, but did not markedly affect specificity and overdiagnosing SIBO. Hence, the 20-ppm cutoff was used for further diagnostics, in line with the American consensus [6].

The ROC and performance analysis showed values for the three cutoffs (12, 15, and 20 ppm) to be close. To decide which cutoff to use, we considered specificity as the most important reason why some degree of sensitivity was lost. Next, we considered positive prediction to be more important as to why the negative predictive value had to be given. However, not all patients with risk factors for SIBO have a clinically relevant SIBO, which is reflected by 9 out of 39 (23%) of our subjects who were predisposed to SIBO but showed breath hydrogen levels within normal limits. The diagnostic accuracy of 81% and 84% for the SIBO-predisposed and all IBS groups in our setting was better than previously reported with 55% accuracy compared to the jejunal bacterial aspirate in the Rome consensus report [17]. A later review of pooled LHBT data showed a sensitivity of 62% and a specificity of 86% [18], while other studies verified a performance similar to ours, with a sensitivity of 86% and a specificity of 77% [19]. These differences may be due to increased awareness of using an optimal time frame for the hydrogen readout, as well as the appropriate cutoff level for diagnosis.

Among the healthy controls, we found a few with high breath hydrogen up to 26 ppm, whereas 11 patients predisposed to SIBO showed values below 20 ppm. Among the patients with IBS, 47 had breath hydrogen levels below 20 ppm, while another 30 showed significantly higher levels. Hence, the IBS cohort was subdivided into low and high hydrogen producers.

Employing the 80-min readout time frame and 20 ppm as the cutoff for the diagnosis of SIBO, we found that 39% of patients clinically diagnosed with IBS were found to have SIBO. This corroborates earlier findings showing that SIBO is frequently found in patients diagnosed with IBS, in the range of 30%–85% [20–24]. To verify this, we used antibiotics in patients with IBS, resulting in a therapeutic response in which 28 of 30 normalized their hydrogen production. The choice of antibiotics was based on the assumptions of pathogenic bacteria in each individual case. Our data showed that high-hydrogen IBS could be treated with symptomatic relief, which is in line with previous experience [22], but with no further information on the recurrence rate of SIBO. However, a significant reduction of the hydrogen production would be expected in all controls and all patients after this form of treatment, why the comparison only confirms that the LHBT is sensitive to this intervention and treatment requires additional relief of symptoms associated with SIBO.

This finding suggests the pathophysiological importance of SIBO in IBS-like symptoms. Along these lines, Esposito et al. proposed the use of LHBT to distinguish SIBO from IBS [25]. Hence, because SIBO is a complex condition that often includes malnutrition and weight loss, personalized treatment is required on a case-by-case basis.

### 4.1. Strengths and Weaknesses

Patients were randomly selected from a clinical cohort based on a recent diagnosis of SIBO to determine whether differences could be observed between the study groups. Anamnestic symptom records may be prone to recall bias, despite efforts to clarify cases in which doubtful clinical signs were provided.

We included healthy volunteers and patients of similar ages with gastrointestinal symptoms, suggesting SIBO. As aging may be important for the development of SIBO associated with chronic diarrhea, malabsorption, weight loss, and secondary nutritional deficiencies [26, 27], our data are normative for the common population and comparable across the study groups.

Lactulose was employed in this study because it travels unchanged throughout the small intestine, enhancing the sensitivity to detect bacterial overgrowth in both proximal and distal regions of the gut. However, a rapid transit time could be misdiagnosed as SIBO if it reaches the colon sooner than the 80-min cutoff time, as shown by scintigraphy. Thus, we advocate a cutoff time of 80 min to avoid false positives, while the European cutoff timing standard goes down to 60 min to avoid false positives [7], however, at the cost of false negatives.

Our study has some limitations. In the preparatory scintigraphic transit study, the radioactive marker was not delivered in the duodenum at exactly the same time as lactulose. To minimize this difference, the starting point for the readout of radioactive and lactulose markers was linked to the best possible alignment. Furthermore, bacterial metabolism in the cecum required to produce hydrogen further delayed the lactulose transit readout. We estimated a 19-min difference between the transit time readouts, which is why a diagnostic time frame of 0–80 min for SIBO was used to mitigate the risk of false positives. As an alternative to the LHBT, the glucose breath test can be used for the detection of SIBO but is less frequently positive in IBS patients, which is why these investigations are not interchangeable for the diagnosis of SIBO [28].

In conclusion, our study supports that the LHBT is capable of diagnosing SIBO, provides a readout time frame, and verifies peak hydrogen cutoff levels for diagnosis. Furthermore, IBS patients can be subdivided into those with and without SIBO. This opens therapeutic possibilities in those suffering from SIBO.

## Figures and Tables

**Table 1:** Lactulose breath test receiver operating characteristics and test performance at different diagnostic cutoff levels.

**Comparison**	**Sensitivity (%)**			**Specificity (%)**			**Likelihood ratio**			**Positive predictive value (%)**			**Negative predictive value (%)**			**Accuracy (%)**		
**Diagnostic comparisons**	**12**	**15**	**20**	**12**	**15**	**20**	**12**	**15**	**20**	**12**	**15**	**20**	**12**	**15**	**20**	**12**	**15**	**20**
SIBO predisposed vs. healthy controls	97	90	77	82	85	88	5.5	6.0	6.3	74	76	77	98	94	88	87	86	84
All IBS vs. healthy controls	44	40	39	72	85	78	2.5	2.7	3.2	72	74	77	98	92	84	85	84	81

Bold numbers show diagnostic comparison across different cutoffs using 12,15, and 20 ppm.

## Data Availability

The data underlying this presentation is available as pseudonymized human data through the guarantor of the article Prof. P.M.H, Department of Medical Sciences, Uppsala University, Uppsala, Sweden. The data is available upon request from the guarantor.
